# Arrhythmogenic Left Ventricular Cardiomyopathy: A Clinical and CMR Study

**DOI:** 10.1038/s41598-019-57203-2

**Published:** 2020-01-17

**Authors:** Jian He, Jing Xu, Guozhong Li, Di Zhou, Shuang Li, Baiyan Zhuang, Xiuyu Chen, Xuejin Duan, Li Li, Xiaohan Fan, Jinghan Huang, Gang Yin, Yong Jiang, Yang Wang, Shihua Zhao, Minjie Lu

**Affiliations:** 1 Department of Magnetic Resonance Imaging, Fuwai Hospital, National Center for Cardiovascular Diseases, State Key Laboratory of Cardiovascular Disease, Chinese Academy of Medical Sciences and Peking Union Medical College, Beijing, China; 2 Department of Radiology, The Second Hospital of Hebei Medical University, Shijiazhuang, Hebei China; 3 Key Laboratory of Cardiovascular Imaging, Chinese Academy of Medical Sciences, Beijing, China; 4 Department of Pathology, State Key Laboratory of Cardiovascular Disease, Fuwai Hospital, National Center for Cardiovascular Diseases, Chinese Academy of Medical Sciences and Peking Union Medical College, Beijing, China; 5 Department of Cardiology, State Key Laboratory of Cardiovascular Disease, Fuwai Hospital, National Center for Cardiovascular Diseases, Chinese Academy of Medical Sciences and Peking Union Medical College, Beijing, China; 6 The Heart-lung Testing Center, State Key Laboratory of Cardiovascular Disease, Fuwai Hospital, National Center for Cardiovascular Diseases, Chinese Academy of Medical Sciences and Peking Union Medical College, Beijing, China; 7 Department of Echocardiography, State Key Laboratory of Cardiovascular Disease, Fuwai Hospital, National Center for Cardiovascular Diseases, Chinese Academy of Medical Sciences and Peking Union Medical College, Beijing, China; 8 Medical Research & Biometrics Center, Cardiovascular Institute and Fuwai Hospital, National Center for Cardiovascular Diseases, Chinese Academy of Medical Sciences and Peking Union Medical College, Beijing, China

**Keywords:** Cardiology, Cardiomyopathies

## Abstract

The clinical features, CMR characteristics and outcomes of arrhythmogenic left ventricular cardiomyopathy (ALVC), which is a very rare nonischemic cardiomyopathy, are currently not well studied. The purpose of the study is to investigate the clinical and cardiovascular magnetic resonance (CMR) imaging characteristics of arrhythmogenic left ventricular cardiomyopathy (ALVC). Fifty-three consecutive patients with ALVC were divided into two groups: ALVC patients without right ventricular (RV) involvement (n = 36, group 1) and those with RV involvement (n = 17, group 2). Clinical symptoms, cardiac electrophysiological findings, and CMR parameters (morphology, ventricular function, and myocardial fibrosis and fatty infiltration) were evaluated in both groups. The two groups showed no significant difference in age, gender, or presenting symptoms (P > 0.05). Right bundle branch block ventricular arrhythmia was less common in patients without RV involvement (50.0% vs.64.7%, P = 0.031). There were no significant differences in left ventricular function between the two groups, however right ventricular ejection fraction was significantly lower in group 2 (40.1 ± 4.0% vs. 48.7 ± 3.9%, P < 0.001). Inverse correlations of left ventricular ejection fraction with fat volume (r = −0.883, p = 0.001), late gadolinium enhancement (LGE) volume (r = −0.892, 0.013), ratio of fat/LGE (r = −0.848, p < 0.001), indexed left ventricular end diastolic volume (r = −0.877, p < 0.001) and indexed left ventricular end systolic volume (r = −0.943, p < 0.001) were all significant. ALVC is a rare disease with fibro-fatty replacement predominantly in the left ventricle, impaired left ventricular systolic function, and ventricular arrhythmias originating from the left ventricle. ALVC with right ventricular involvement may have a worse prognosis.

## Introduction

Arrhythmogenic right ventricular cardiomyopathy (ARVC) is a rare cardiomyopathy with a prevalence of 1/1000 to 1/5000^1^. It has been described as an inherited cardiomyopathy characterized by progressive fibro-fatty or fatty replacement of the right ventricular (RV) myocardium resulting in abnormalities of RV morphology and function^2–5^. Left ventricular function is often preserved at early stages of ARVC and even with end-stage disease, left ventricular morphology and function are much less affected than the right ventricle^4,6^. However, cases of isolated left ventricular (LV) fibro-fatty infiltration/replacement also exist and although these cases may have some overlapping features with ARVC, there are also many different features and these cases should not be referred to as ARVC^7–9^. Patients with left ventricular myocardial fibro-fatty disease usually complain symptoms of both arrhythmia (palpitations, chest tightness, syncope, etc.)^10,11^ and LV heart failure^12^. Imaging modalities usually reveal severe LV dysfunction with preserved to mildly impaired RV function. Often this entity has been misdiagnosed as dilated cardiomyopathy, chronic myocarditis, myocardial infarction, or double ventricular involvement type ARVC^12,13^. Although there have been a handful publications detailing this rare disease, the majority of these have been case reports^7,13–15^, and both the clinical cardiac magnetic resonance (CMR) characteristics remain poorly studied and consistent nomenclature for this cardiomyopathy is not present. It has been referred to as both arrhythmogenic left ventricular cardiomyopathy(ALVC)^7,12,16^ and desmosomal cardiomyopathy^17^. ALVC has also been referred to as arrhythmogenic cardiomyopathy with left ventricular involvement^11^.

The aim of the present study is to report the frequency, clinical manifestations, and CMR characteristics of this rare cardiomyopathy with predominantly left ventricular fatty or fibro-fatty infiltration. We also hope to improve the clinical awareness of this disease, which will be referred to as arrhythmogenic left ventricular cardiomyopathy (ALVC) in this manuscript, and to provide information that will aid in both clinical decision making and further management of this group of patientss.

## Methods

### Subjects

We systematically evaluated 35,845 patients who were referred for CMR examinations at Fuwai Hospital (Beijing, China) between 2004 and 2017. Patients with arrhythmogenic cardiomyopathy who had predominantly left ventricular fibro-fatty involvement were retrospectively collected according to clinical criteria mainly from Dr. Sen-Chowdhry’s study^11^ including 7 different criteria in 4 categories and (1. Arrhythmia: Sustained or nonsustained ventricular tachycardia; 2. Imaging: 1) LV aneurysms, 2) Mild LV dilation and/or systolic impairment ;3. Biopsy/CMR: 1)Myocyte loss with fibrofatty replacement on histology, 2).Extensive LGE of LV myocardium (with subepicardial/midmyocardial distribution); 4. ECG: Unexplained T-wave inversion in V5, V6 ± V4, I, and aVL. All the patients must meet at least the first three of the four categories of diagnostic criteria, so the minimum requirement of inclusion criteria was 3 criteria in the last 3 categories, up to 7 criteria in 4 categories).

Patients were excluded if they had any of the following: (1) Patients who meet the definite diagnostic criteria for ARVC^5^; (2) Patients with other nonischemic cardiomyopathies including hypertrophic cardiomyopathy, dilated cardiomyopathy, restrictive cardiomyopathy and myocarditis;(3) coronary artery disease (stenosis >50% of the luminal diameter in a major branch) or myocardial infarction; (4) congenital heart disease, (5) moderate or severe valvular heart disease; (6) sustained, rapid, uncontrolled supraventricular arrhythmia, or (7) a contraindication to CMR scanning.

This study was approved by the Ethics Committee of Fuwai Hospital and it complies with the ethical principles of the Declaration of Helsinki. Written informed consent was waived by the Ethics Committee of Fuwai Hospital because this study was retrospective.

### CME protocols

All CMR exams were performed on one of three MR scanners: 1.5 T Magneton Avanto (Siemens Healthcare), 3.0 T MR750 (GE Healthcare), and 3.0 T Ingenia (Philips). All patients were imaged in the supine position. The study consisted of morphological and functional imaging including 2D transverse and sagittal imaging as well as short and long axis ventricular cine imaging, LGE imaging, tissue characterization with T1 and T2 weighted imaging and water-fat-separating imaging.

For each imaging sequence, identical slice locations were used. These slices consisted of 8 parallel 6-mm-thick short-axis slices with a 4–6 mm section gap. The slices extended from the base to the apex of the heart. A balanced steady-state free precession (bSSFP) was used for cine imaging. Late gadolinium enhancement (LGE) imaging was performed with a T1-weighted pulse sequence with phase sensitive inversion recovery (PSIR). Intravenous gadodiamide (Magnevist^®^, Bayer and Schering^®^) was administered and a dose of 0.2 mmol/kg was used. LGE images were collected 10–15 minutes after the contrast administration. Gadolinum contrast was administered with an automated injector (Spectris^®^; Medrad^®^, Pittsburgh, Pa).

A multi-echo GRE sequence was implemented with fat and water separation using the (variable projection) VARPRO multi-point Dixon reconstruction method^18^ with T2* correction^19^. Typical parameters were: bandwidth = 930 Hz/pixel, TE = 1.35, 3.10, 4.85, and 6.8 ms, TR = 374.25 ms, flip angle = 24°, image matrix = 192 × 112, views-per-segment = 5, breath-hold duration = 16 heartbeats including 2 initial heartbeats discarded for transition to steady state. The inversion time was auto-adjusted to minimize the signal intensity of normal myocardium.

### Image analysis

All images were analyzed offline using a workstation with commercially available software (Argus® Version 3.3, Siemens®, Germany) and Medis^®^ Version 5.0 (Medis, Netherlands). For all patients, the CMR scans were anonymised and placed in a random order for interpretation. Interpreting physicians evaluating the CMR exams were blinded to the clinical data. Dimensions of both atria and ventricles were measured as previously described^20,21^. End-diastole (ED) and end-systole (ES) were visually determined. Semi-automatic tracing with manual correction of epicardial and endocardial borders of contiguous short-axis left ventricular slices was performed to measure ventricular mass (VM), end-diastolic volume(EDV), and end-systolic volume (ESV). The EDV and ESV were used to derive the ejection fraction (EF), cardiac output (CO) and cardiac index(CI). The LVM was calculated by subtracting endocardial from epicardial volume at end diastole and multiplying by 1.05 g/cm^3^. All the global functional parameters were indexed to body surface area.

Hyper-enhanced pixels were defined as LGE if the signal intensities were 5 standard deviations greater than the mean signal intensity within nulled, remote myocardial region on the same image. The 17-segment AHA model was used to categorize areas of LGE into corresponding LV segments. The amount of LGE was calculated into mass. A fat fraction(FF) map (Equation [1]) was used to determine the presence of fat^22^ by using the fat only (F) and water only (W) images. Before doing the fat fraction map processing, a magnitude discrimination was performed to correct bias from T1 and noise^22,23^ and fat pixels were defined as higher than 50% in the fraction map^24^.

1 $${\rm{FF}}={\rm{F}}/({\rm{W}}+{\rm{F}})\ast 100 \% $$

All image analyses were performed by a single investigator with 13 years of cardiovascular MRI analysis experience. To assess the reproducibility, LGE and fat volumes were performed by 2 independent experienced interpreters who had 13 years and 9 years of cardiovascular MRI analysis experience.

### Statistical analysis

Continuous variables with normal distribution are summarized as the mean ± standard deviation and data not fulfilled with normal distribution were presented as median (Q_25_, Q_75_). Comparisons between continuous variables determined in the subgroups of patients included in the study were performed by using the student *t* test. Linear correlation was used to evaluate the correlation indices (Pearson coefficient, *r)* between LVEF and fat, and LVEF and LGE, as well as between LVEF and LV mass index. Interobserver variability was assessed using the Bland -Altman method^25^.

Categorical variables are presented as a frequency or a percentage and were compared via the Fischer exact test. A multiple regression model was used to analyze the independent predictive values of fat deposition and LGE volumes on global cardiac functional variables. For survival analysis, Kaplan-Meier survival curves were compared using log-rank statistics. We use a combined end-point including death from non-cardiovascular disease, heart transplantation, heart failure death and sudden cardiac death^26^. All outcome events were reviewed by two independent investigators, using previously described criteria^27^. For each test applied in this study, *P* values of 0.05 or less were considered to indicate significance. All statistical analyses were performed by using software (SPSS version 13.0; SPSS, Chicago, IL) and GraphPad Prism statistical software package (GraphPad Software, San Diego, CA, Version 5.01).

## Results

### Patient characteristics

**Figure 1:**
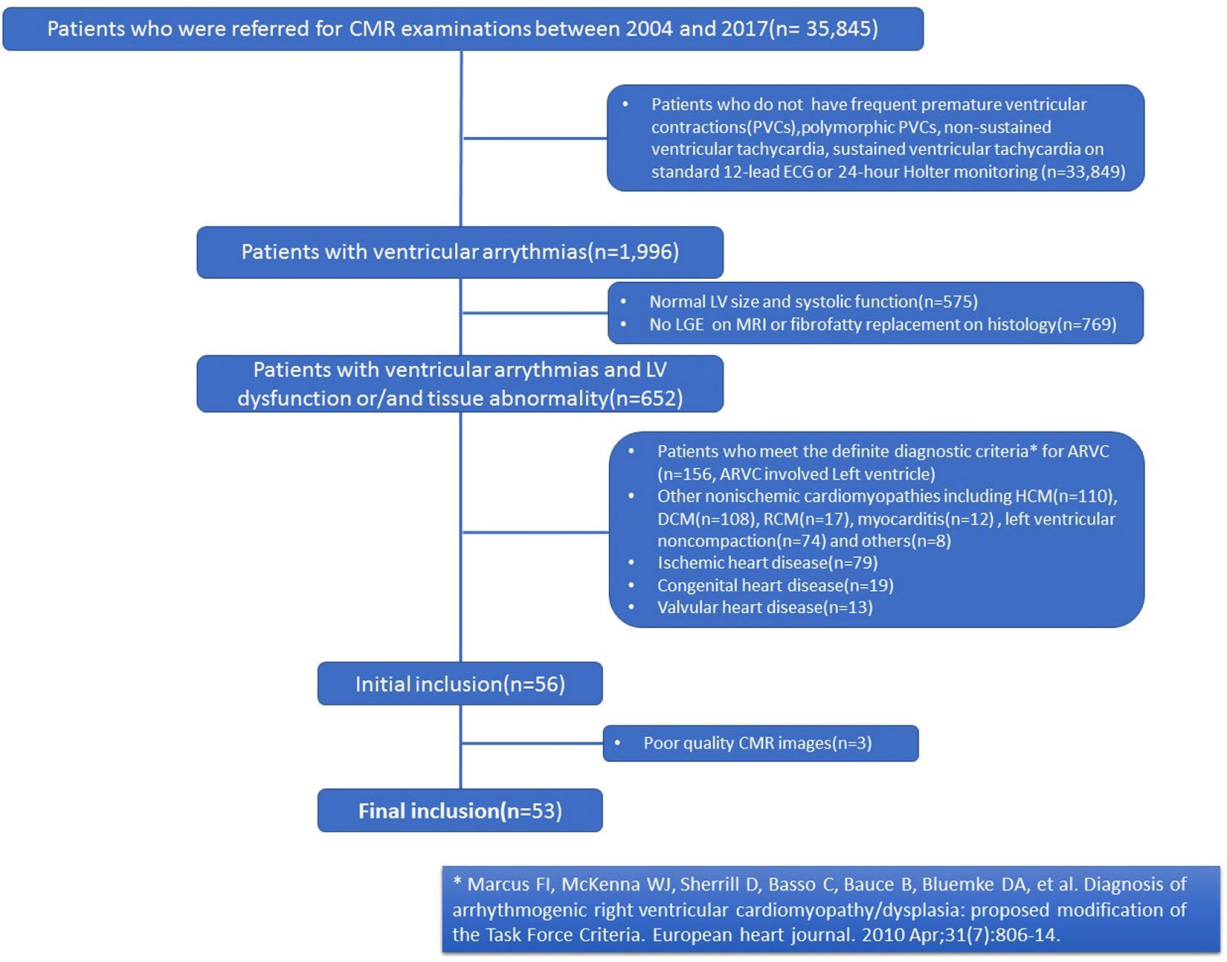
The flowchart shows the patient selection process based on inclusion and exclusion criteria detailed in the methods section.

A total of 33,849 patients were primary excluded for not presenting with ventricular arrhythmia. Another 575 patients with normal LV function and 769 with negative LGE were further excluded. Finally, a total of 53 patients(0.16%) from the whole cohort of 35,845 patients fulfilled the inclusion criteria and were included for the analysis in current study (Fig. 1). The detailed characteristics and distribution of the inclusion criteria applied in the current study were presented in supplemental material (Table 1). Of the 53 patients, five patients had biopsy specimen proven diagnosis of ALVC, four patients underwent heart transplantations. The presence of fibro-fatty replacement was confirmed by endomyocardial biopsy in five patients and by heart transplantation in all four patients. The patients were divided into 2 groups: patients with No RV involvement (n = 36) and patients with RV involvement (n = 17). Involvement of the right ventricle was determined by either local/global dysfunction +/− fat/fibrosis in right ventricle. Patient demographic and clinical data is summarized in Table 2.

**Table 1:** Detailed distribution of inclusion criteria for all patients in this cohort (n = 53).

Subgroup	ECG^*^	Arrhythmia^@^		Imaging^#^		Tissue Characteristics^$^		Total number of inclusion criteria meets				
		a	b	c	d	e	f	7	6	5	4	3
LV alone (n = 36)	21	8	36	3	36	5	36	0	1	9	16	10
Bi-ventricular (n = 17)	13	6	17	3	17	4	17	0	2	5	10	0

*ECG: Unexplained T-wave inversion in V5, V6 _ V4, I, and aVL; ^@^Arrhythmia. Ventricular arrhythmia, ^#^Imaging. CMR SSFP cine; ^$^Biopsy/CMR. Myocardial fat-fibrosis replacement by endocardial biopsy, heart transplantation and CMR characteristics; a. VT. Sustained or nonsustained ventricular tachycardia; b. PVCs. Frequent ventricular extrasystoles; c. LV LV dilation; d. LV systolic impairment; e. Biopsy/HT: endocardial biopsy and heart transplantation; f. CMR: Tissue characteristics by comprehensive CMR techniques including turbo spin echo T1/T2 weighted imaging, water/fat separation and late gadolinium enhancement.

**Table 2:** Demographic and Clinical Profile of this Cohort.

	Patients (n = 53)	No RV involvement (n = 36)	RV involvement (n = 17)	*P*
Gender(Male/Female)		24/12	8/9	0.700
Age	40.3 ± 12.1	40.8 ± 12.5	39.5 ± 11.5	0.646
BMI	21.9 ± 2.2	22.1 ± 2.3	21.5 ± 2.2	0.402
Symptoms				
asymptomatic	7(13.2)	5(13.9)	2(11.8)	0.833
palpitation	39(73.6)	22(61.1)	13(76.5)	0.275
chest pain	5(9.4)	3(8.3)	2(11.8)	0.693
chest tightness	10(18.9)	5(13.8)	5(29.4)	0.182
exertional dyspnea	21(39.6)	15(41.7)	6(35.3)	0.661
syncope	12(22.6)	7(19.4)	5(29.4)	0.423
Family history of sudden cardiac death	5(9.4)	2 (5.6)	3(17.6)	0.164
NYHA				0.524
I	20(37.7)	14(38.9)	6(35.3)	
II	20(37.7)	14(38.9)	6(35.3)	
III	9(17.0)	7(19.4)	2(11.8)	
IV	4(7.5)	1(2.8)	3(17.6)	
12-lead ECG abnormalities				
T-wave inversion	34(64.2)	21(58.3)	13(76.5)	0.203
Ventricular premature beat from LV	41(77.4)	27(75.0)	15(88.2)	
Ventricular arrhythmia of RBBB morphology	29(54.7)	18(50.0)	11(64.7)	0.031
Ventricular arrhythmia of LBBB morphology	20(37.7)	10(27.8)	10(58.8)	0.320
Atrial premature beat	4(7.5)	4(11.1)	0	0.157
Holter				
Premature ventricular beats <1000/24 h	9(17.0)	7(19.4)	2(11.8)	0.618
Premature ventricular beats >1000/24 h	45(84.9)	30(83.3)	15(88.2)	0.618
polymorphic PVCs	23(43.4)	13(36.1)	10(58.8)	0.096
NSVT	23(43.4)	13(36.1)	10(58.8)	0.486
SVT	1(1.9)	1(2.8)	0	0.486
Medication				
None	5(9.4)	4(11.1)	1(5.9)	0.238
β-blockers	36(67.9)	21(58.3)	15(88.2)	0.031
Amiodarone	23(43.4)	13(36.1)	10(58.8)	0.123
ACE-inhibitor	9(17.0)	4(11.1)	5(29.4)	0.101
Diuretic	5(9.4)	4(11.1)	1(5.9)	0.547
Others	7(13.2)	3(8.3)	4(23.5)	0.131

ALVC: arrhythmogenic left ventricular cardiomyopathy; ARVC: Arrhythmogenic left ventricular cardiomyopathy; DCM: dilated cardiomyopathy; HCM: hypertrophic cardiomyopathy; RCM: restrictive cardiomyopathy; NYHA: New York Heart Association; RBBB: right bundle branch block; LBBB: left bundle branch block; PVCs: Premature ventricular contractions; NSVT: nonsustained ventricular tachycardia; SVT: sustained ventricular tachycardia.

There was no statistically significant difference between the two subgroups in the majority of clinical characteristics with the exception of β-blocker treatment. Patients who were in the group without RV involvement of disease were more likely to be on a β-blocker. Five patients (9.4%) had a family history of sudden cardiac death. The most common clinical presentations were palpitations (73.6%) and exertional dyspnea (39.6%). Twelve of 53 patients (22.6%) presented with syncope as the onset symptom. Beta-blockers were used in 67.9% of the patients. Twenty-three patients with systolic impairment and nine patients with severe ventricular arrhythmia were on angiotensin-converting enzyme inhibitors and amiodarone, respectively.

### ECG and 24-hour Holter Findings

All patients underwent standard 12-lead ECG and 24-hour Holter examinations. The detailed results were summarized in Table 2. The main abnormalities on the 12-lead ECG were premature ventricular beats from the left ventricle (77.4%) followed by T-wave inversion and ventricular arrhythmia of RBBB morphology. There was no statistically significant difference between the prevalence of these 12-lead ECG findings between the two groups. The 24-hour Holter documented 23 patients (43.4%) with nonsustained ventricular tachycardia, 45 patients (84.9%) with frequent premature ventricular beats (ie, >1,000 over 24 hours of Holter monitoring), and 23 patients (43.4%) with multi-source premature ventricular beats. There was no statistically significant difference between the prevalence of these 24-hour Holter findings between the two groups.

### CMR findings

**Figure 2:**
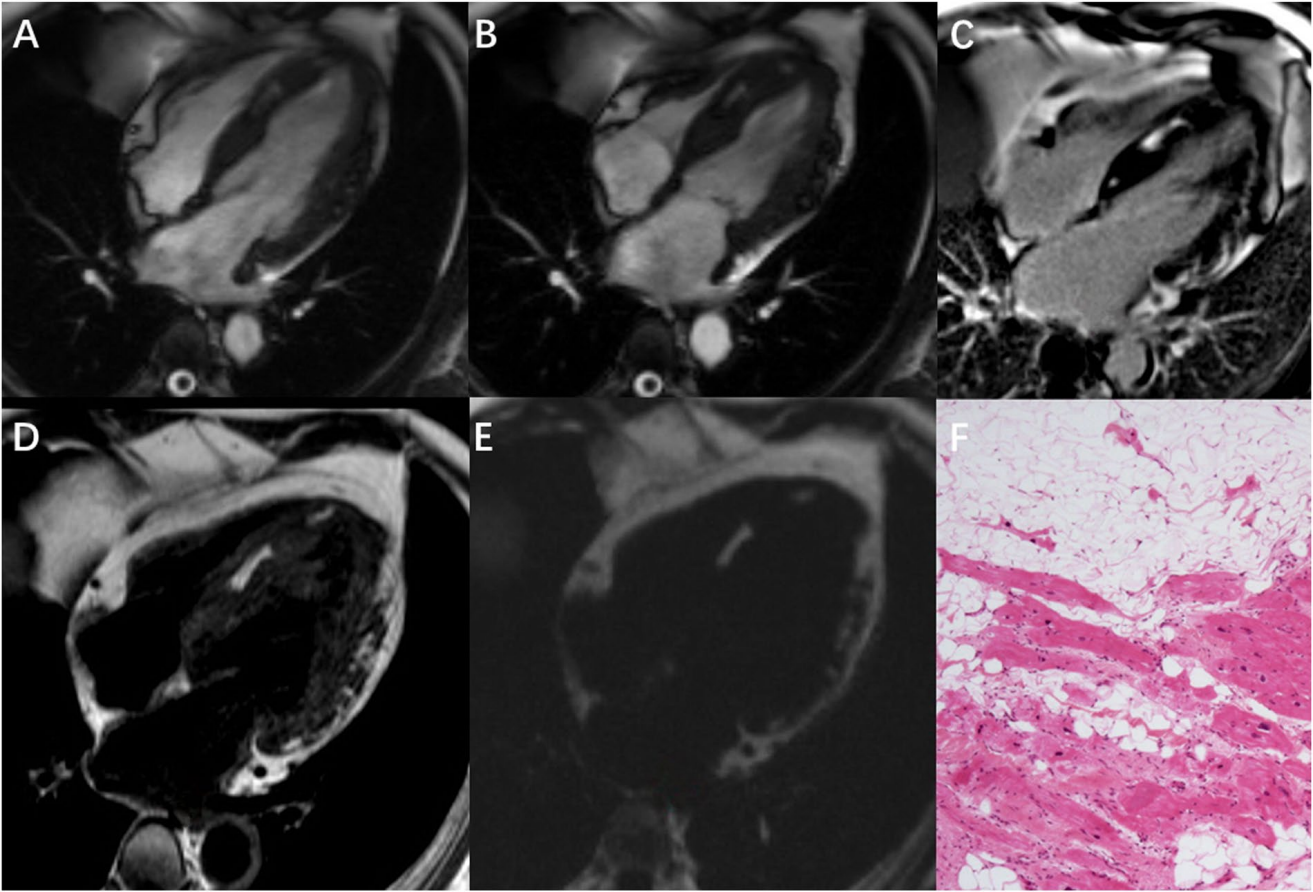
A typical case of arrhythmogenic left ventricular cardiomyopathy in a 54 year-old male patient with frequent ventricular premature beats and syncope. Four chamber view of (**A**) End-diastolic SSFP cine, (**B**) End-systolic SSFP cine, (**C**) Late gadolinium enhancement imaging (LGE) (**D**) T1 weighted image and (**E**) fat image from water-fat separation imaging all show fibro-fatty infiltration in the interventricular septum and the epicardial LV lateral wall. Note that the contour of the lateral LV wall is irregular with a “serrated” shape. LGE shows significantly delayed enhancement of the LV basal to mid lateral wall, interventricular septum and adjacent anterior wall. (**F**) Histology of endomyocardial biopsy shows areas of fibro-fatty infiltration and replacement of the myocardium.

**Figure 3:**
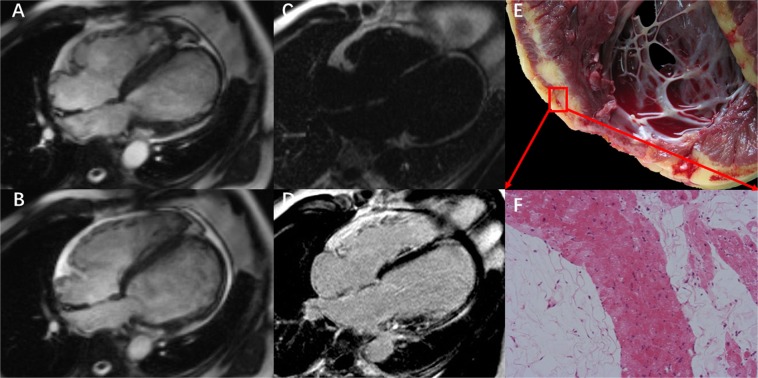
A typical case of arrhythmogenic left ventricular cardiomyopathy with right ventricular involvement in a 31 year-old male with dyspnea on exertion and syncope. Four chamber SSFP cine images during (**A**) diastole and (**B**) systole show that the left ventricle is significantly dilated(LVEDD 65 mm) and there is also mild to moderate right ventricular dilation. The wall of the LV apex is thin and aneurysmal. (**C**) fat image from the water-fat separation imaging shows fat within the left ventricular walls. (**D**) 4 chamber LGE image shows significant enhancement within the LV basal to mid lateral wall, interventricular septum, and LV apex. (**E,F**) Histology of the explanted heart showed severe fibro-fatty infiltration and replacement of the left ventricular wall.

The detailed results of the CMR imaging study were summarized in Table 3. CMR detected structural and functional LV abnormalities in all 53 patients (Fig. 2) including the 17 patients (32.1%) with RV involvement (Fig. 3). With the exception of RV ejection fraction (RVEF) and RV end-systolic volume index (RVESVI), there was no significant difference between the two groups with regards to functional parameters.

**Table 3:** CMR parameters of ALVC with further subgroups analysis.

CMR Parameters	Total (n = 53)	No RV involved (n = 36)	RV involved (n = 17)	t	*P*
LAD(mm)	40.4 ± 3.3	40.7 ± 3.1	39.7 ± 3.7	1.016	0.314
LVEDD (mm)	65.2 ± 5.6	65.0 ± 5.2	66.8 ± 6.3	−1.101	0.276
LVOTD(mm)	32.2 ± 4.3	32.4 ± 4.6	30.7 ± 3.1	1.354	0.182
LVEF(%)	41.5 ± 4.5	41.7 ± 4.1	41.1 ± 5.3	0.412	0.682
LVEDVi(ml/m^2^)	95.5 ± 14.6	94.5 ± 13.5	97.6 ± 16.9	−0.711	0.480
LVESVi(ml/m^2^)	56.5 ± 12.5	55.6 ± 11.3	58.3 ± 15.0	−0.715	0.478
LVSV(ml)	67.8 ± 8.9	68.0 ± 8.6	67.2 ± 9.6	0.296	0.768
LVCI(L/min/m^2^)	2.82 ± 0.40	2.78 ± 0.39	2.91 ± 0.39	−1.174	0.246
LV Mass Index(g/m^2^)	59.9 ± 10.2	58.7 ± 9.2	62.3 ± 11.9	−1.187	0.241
LV Fat mass(g)	4.27 ± 2.85	4.00 ± 2.49	4.84 ± 3.52	−0.999	0.323
LV Fat percent(%)	3.91 ± 2.25	3.70 ± 1.90	4.35 ± 2.88	−0.979	0.332
LV LGE mass (g)	8.24 ± 3.49	8.05 ± 3.19	8.66 ± 4.12	−0.592	0.556
LV LGE percent(%)	7.78 ± 2.59	7.69 ± 2.32	7.97 ± 3.16	−0.359	0.721
LV Fat/LGE	0.47 ± 0.15	0.45 ± 0.14	0.50 ± 0.16	−1.162	0.251
RAD(mm)	44.2 ± 3.8	45.0 ± 3.6	42.8 ± 4.0	1.977	0.053
RVEDD(mm)	26.3 ± 4.9	26.4 ± 4.8	26.1 ± 5.3	−1.162	0.251
RVOTD(mm)	24.4 ± 3.8	24.1 ± 3.8	25.1 ± 3.7	−0.898	0.374
RVEF(%)	45.9 ± 5.6	48.7 ± 3.9	40.1 ± 4.0	7.477	<0.001
RVEDVi(ml/m^2^)	78.4 ± 11.7	75.3 ± 11.8	77.7 ± 11.6	−0.720	0.475
RVESVi(ml/m^2^)	41.2 ± 7.9	75.3 ± 11.8	77.7 ± 11.6	−3.784	<0.001
RVSV(ml)	60.1 ± 10.9	63.5 ± 9.5	53.0 ± 10.4	3.634	0.001
RVCI(L/min/m^2^)	2.51 ± 0.50	2.60 ± 0.47	2.32 ± 0.54	1.933	0.059
RV Mass Index(g/m^2^)	27.4 ± 6.1	27.4 ± 6.68	27.4 ± 4.68	0.037	0.970
RV Fat mass (g)	0(0,1.4)	0	2.2 ± 0.8	−16.401	<0.001
RV Fat percent(%)	0(0,3.18)	0	4.81 ± 2.03	−9.764	<0.001
RV LGE mass (g)	0(0,2.6)	0	3.2 ± 1.0	−12.938	<0.001
RV LGE percent(%)	0(0,5.65)	0	7.05 ± 2.65	−10.975	<0.001
RV Fat/LGE	0(0,53.47)	/	0.70 ± 0.20	−14.027	<0.001

Continuous variables with normal distribution were presented with mean ± SD and data not fulfilled with normal distribution were presented with median (Q_25,_ Q_75_). LAD: Dimension of left atrium; LVEDD: left ventricular end-diastolic dimension; LVOTD: left ventricular outlet tract dimension; LVEF: left ventricular ejection fraction; LVEDVi: left ventricular end-diastolic volume index; LVESVi: left ventricular end-systolic volume index; LVSV: left ventricular stroke volume; LVCI: left ventricular cardiac index; LV: left ventricular; LGE: late gadolinium enhancement; RAD: Dimension of right atrium; RVEDD: right ventricular end-diastolic dimension; RVOTD: right ventricular outlet tract dimension; RVEF: right ventricular ejection fraction; RVEDVi: right ventricular end-diastolic volume index; RVESVi: right ventricular end-systolic volume index; RVSV: right ventricular stroke volume; RVCI: right ventricular cardiac index; RV: right ventricular.

**Figure 4:**
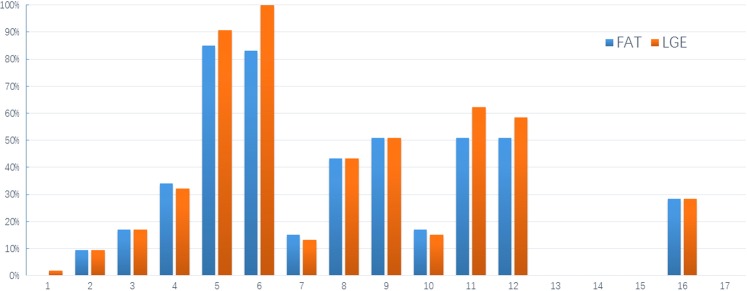
Bar graph of AHA 17-segment model demonstrating the segments with LGE and fat (Segment 17 was not analyzed).

**Figure 5:**
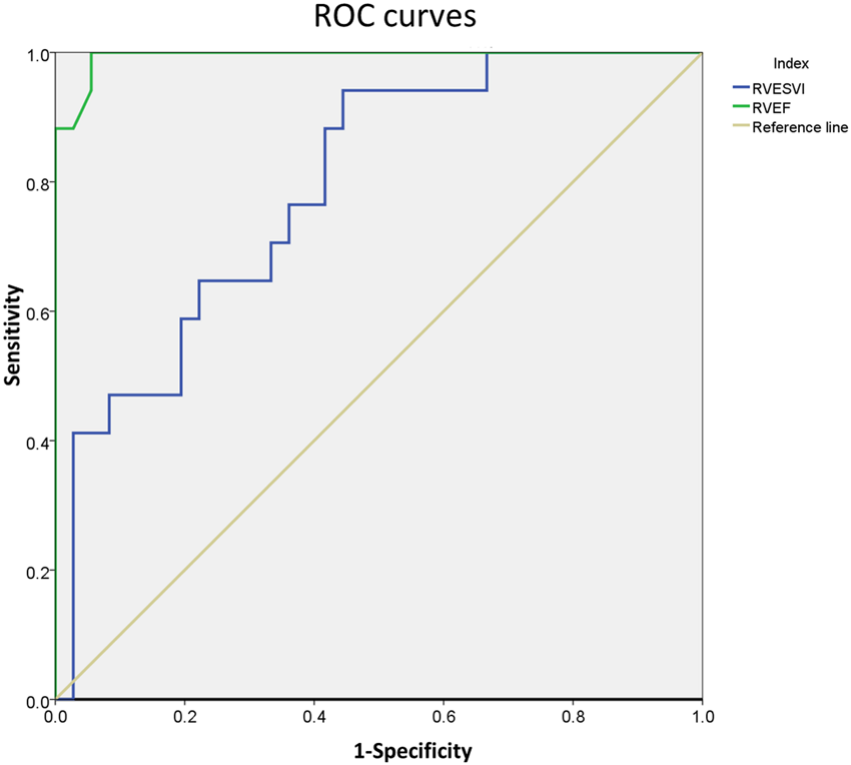
Receiver operating curves showing the predictive performance of RVESVi and RVEF in the differentiation of RV involvement in patients with ALVC. The RVEF has the larger area under curve when the threshold was 44.1%, with a sensitivity of 100% and a specificity of 94.4%. ROC = receiver operating curve; RVESVi = Right ventricular end-systolic volume index, RVEF = right ventricular ejection fraction.

Intramyocardial fatty infiltration of the LV was observed in 53 patients (100%). The most common location of fatty infiltration was the basal inferolateral wall (85.9%) followed by basal anterolateral wall (83.0%), mid inferoseptal wall (50.1%), midinferolateral wall (50.1%) and mid anterolateral wall (50.1%). LGE of the LV was observed in all 53 patients. The area of LGE overlapped with the areas that contained fat. Quantitative analysis of fat and LGE showed that the amount of LGE was always greater than the amount of fat both in left and right ventricles, consistent with the presence of both fat and fibrosis within the observed areas of LGE (Fig. 4). The ROC curve demonstrated that RVEF of 44.1% was a very good predictor of right ventricular involvement in ALVC with a sensitivity of 100%, specificity of 94.4%, and a ROC AUC of 0.994 (P < 0.001, Fig. 5).

### Predictors of cardiac function

**Figure 6:**
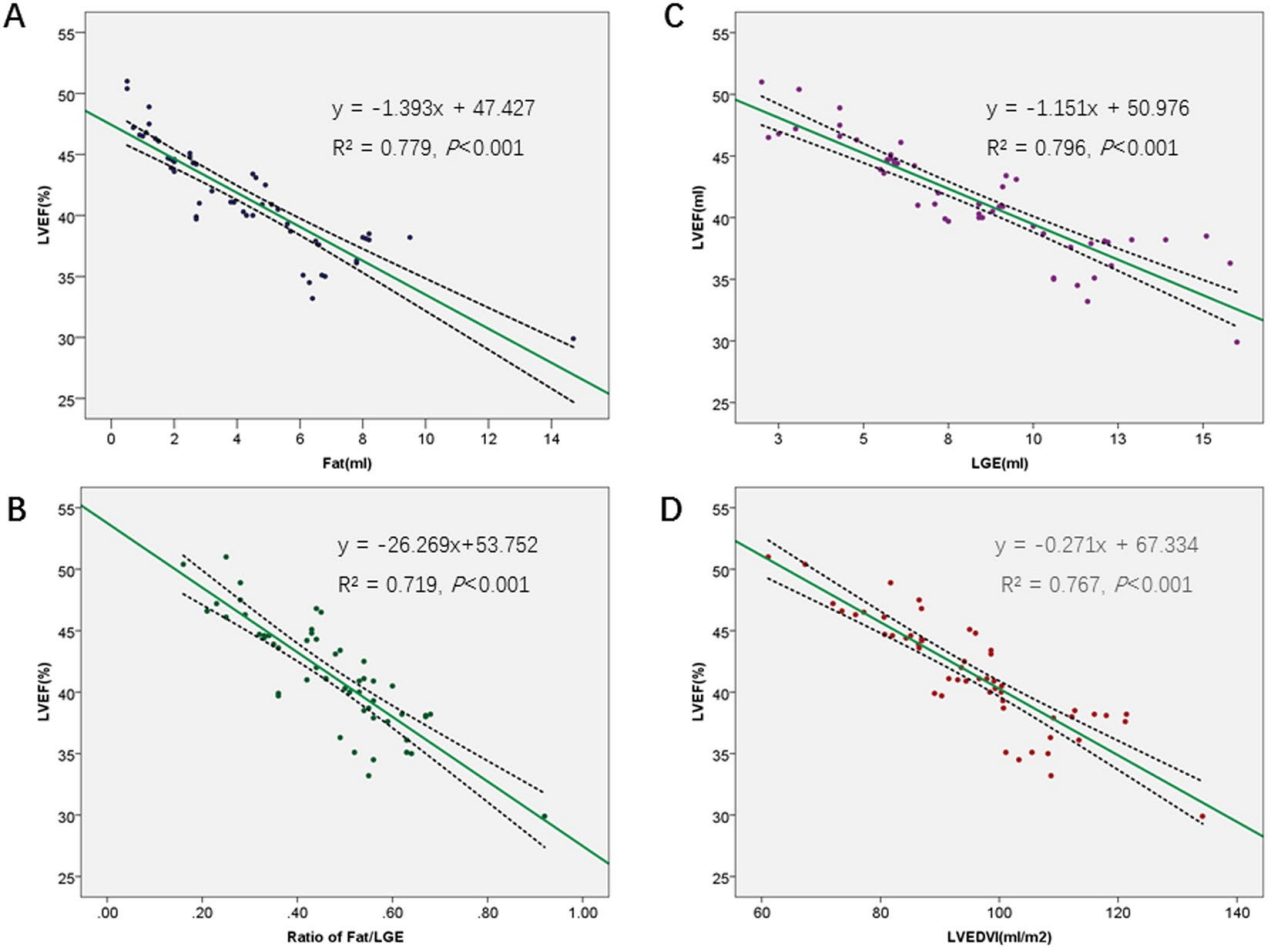
Negative linear relationship between LVEF and (**A**) FAT, (**B**) LGE, (**C**) FAT/LGE and (**D**) LVEDVI.

Since there were no statistical differences in left functional parameters between patients with and without RV involvement, linear correlation was performed in the whole cohort. There was a significant inverse correlation between fat volumes (r = −0.883, p = 0.001), LGE volume(r = −0.892, 0.013), fat/LGE ratio(r = −0.848, p < 0.001), LVEDVI (r = −0.877, p < 0.001) and LVESVI (r = −0.943, p < 0.001) with LVEF (Fig. 6). However, there were no significant univariate correlations between LVEF and dimension of left atrium (LAD, p = 0.103), LVEDD (p = 0.130), and LVSV (p = 0.343). In addition, there was a univariate correlation between fat and fibrosis volumes (r = 0.948, p < 0.001).

### Outcomes

**Figure 7:**
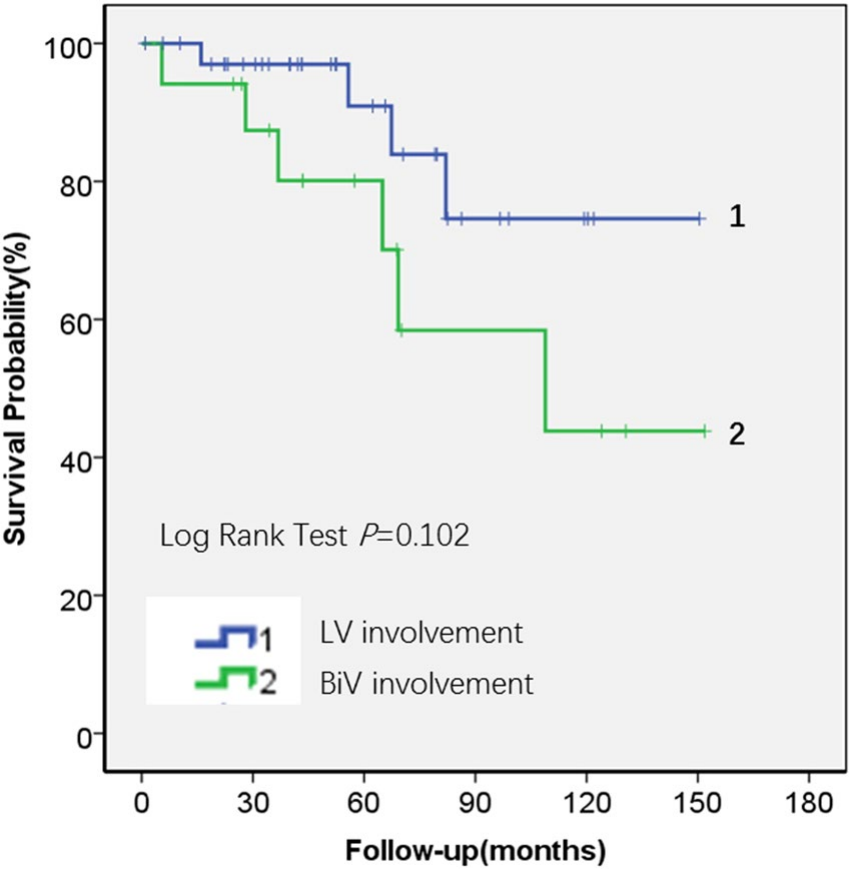
Kaplan-Meier estimates of the proportion of patients with cardiac mortality and heart transplantation free in the ALVC patients with No RV involvement(n = 36) and RV involvement (n = 17).

The mean follow-up periods were 59 months for the whole cohort (56 months for No RV involvement group and 64 months for RV involvement group). During the follow-up period, a total of 10 patients (18.9%) reached primary end points, including 4 patients in the group with No RV involvement (1 heart failure-related death and 3 heart transplantations) and 6 patients (4 sudden deaths, 1 heart transplantation and 1 heart failure-related death) in the RV involvement group. Figure 6 showed Kaplan–Meier survival curves for all-cardiac mortality and heart transplantation free in the two groups. Patients with RV involvement had relatively poor survival, but this result did not reach the statistical significance (Log-rank test: *P* = 0.102, Fig. 7).

### Intra- and Interobserver variability

**Figure 8:**
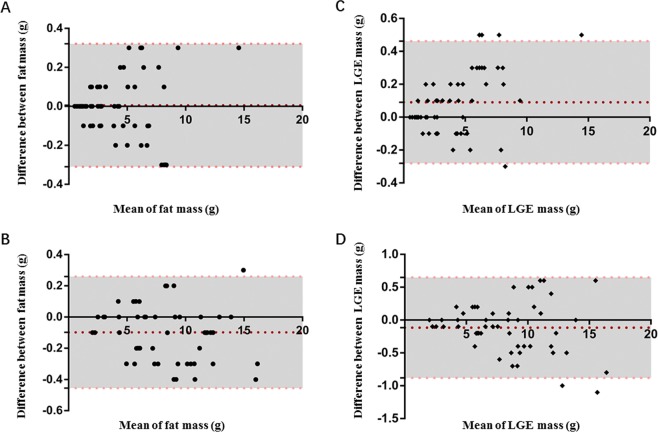
Bland and Altman Analyses of LGE and Fat Volume for intra- and inter-observer Variability. Variability of intra-observer (**A**) and inter-observer (**B**) for fat quantification, intra-observer (**C**) and inter-observer (**D**) for LGE quantification. Red dashed line indicates the mean difference and pink dashed lines indicate SD.

Fat and LGE had an intraobserver variability of 0.01 ± 0.16 g(Median, 0.00 g, Q_25_, −0.10 g, Q_75_,0.10 g) and 0.10 ± 0.18 g (Median, −0.10 g, Q_25_, −0.30 g, Q_75_,0.00 g), and an inter-observer variability of 0.10 ± 0.19 g (Median, 0.10 g, Q_25_, 0.00 g, Q_75_,0.20 g) and 0.12 ± 0.39 g (Median, −0.10 g, Q_25_, −0.40 g, Q_75_,0.10 g), respectively (Fig. 8).

## Discussion

There have been a few reports/studies regarding ALVC in the literature^15–17,28^. Compared with previous studies and case reports, our study is currently the largest cohort of ALVC and has added new insights into this rare cardiomyopathy in terms of clinical features, CMR characteristics and outcomes. Our study yielded several results: 1. ALVC is a very rare disease, accounting for only 0.15% of all the patients who underwent cardiovascular magnetic resonance examinations in our center. 2. In terms of clinical presentation, we found that palpitations and exertional dyspnea were the most common presenting symptoms. Some patients also presented syncope as the initial reason for doctor. Overall, these symptoms are not significantly different from presenting symptoms of ARVC, thus the two entities may be confusing if relying on clinical features only. 3. In regards to electrophysiological abnormalities, there were no statistically significant differences on the standard 12-lead ECG or 24-hour Holter between the group of ALVC with RV involvement and the ALVC group without RV involvement. These findings are in keeping with and further expand findings from Sen-Chowdhry *et al*. who reported detailed ECG finding in a cohort of 42 patients with left-dominant arrhythmogenic cardiomyopathy^11^. In addition, Dr. José María López-Ayala and his colleagues reported a 56%(9 of 16) prevalence of T wave in genetic-positive patients^29^. 4.Typical CMR characteristics of ALVC include left ventricular fatty or fibro-fatty infiltration with LV systolic dysfunction. Some of the patients (32%, 17/53) also had right ventricle fibro-fatty infiltration with mild to moderate RV dysfunction. We can determine whether ALVC has right ventricular involvement by RVEF, and the area under the ROC curve is 0.994.

Furthermore, we also found that an increased amount of fibro-fatty infiltration, increased amount of LGE, and increased Fat/LGE correlated with a worse left ventricular ejection fraction. Although our multiple regression analysis showed that fat, LGE, and LVEDVI/LVESVI were independent predictors of LVEF, the cause and effect remain unknown at this stage and should be investigated in multicenter studies with larger cohorts. In addition, the results of survival analysis showed that patients with the RV involvement had a relatively poorer prognosis at a mean follow-up of 59 months, however this did not reach statistical significance likely due to the relatively small number of patients and short time follow-up period.

ALVC and ARVC can both have biventricular involvement, and CMR exam findings are the key to differentiating these two disease entities^7,11,12^. ALVC has distinctive CMR findings. In our study, we found that the key findings of ALVC are fibro-fatty infiltration of the left ventricle with associated left ventricular dysfunction and arrhythmias from the left ventricle. We also found that when the right ventricle was involved too, left ventricular involvement tended to still be more severe than the right ventricle dysfunction and the ejection fraction of the left ventricle was significantly lower than that of the right ventricle. These findings are similar to what has been previously reported in the literature^28^. This is significantly different compared to end-stage arrhythmogenic RV dysplasia. In end-stage ARVC, the left ventricle can also be involved but compared to the right ventricle it is much less severely affected where the right ventricle is much more severely affected^3,4,30^. Using CMR feature-tracking technique, Dr. Vives-Gilabert *et al*. reported two important mechanisms in arrhythmogenic cardiomyopathy patients with LV involvement including 1) decreased myocardial deformation with global LV affectation and 2) delayed myocardial contraction at localized regions^31^.

It should be noted that ALVC with LV segmental dysfunction can be confused with dilated cardiomyopathy or ischemic cardiomyopathy (chronic myocardial infarction). The prevalence of frequent ventricular arrhythmias reported in the literature was 30–42%^32–34^. Some special gene mutations in dilated cardiomyopathy such as DSP, LMNA, SCN5A, and FLNC have an arrhythmia rate of more than 30%^35^. Dr. Spezzacatene *et al*.^36^ reported that they found up to 38.2% (109/285) of DCM met criteria for arrhythmogenic-DCM phenotype, which is consistent with previous publications. However, the inclusion criteria of that study only consisted of left ventricular ejection fraction assessed by echo and ECG. No myocardial tissue characteristics (fat and myocardial fibrosis) were included in the inclusion criteria. Therefore, it is not possible to exclude the patients with dilated cardiomyopathy caused by idiopathic ventricular arrhythmias. In addition, dilated cardiomyopathy usually does not affect the right ventricle until at the end-stage. However, according to the results of the ROC curve(AUC: 0.994) in current study, the reduction of the RVEF is one of the essential characteristics of ALVC involving the right ventricle. And further survival curves indicate that it is not only an important parameter for differential diagnosis but also for prognostic prediction. Regarding the differentiating from ischemic cardiomyopathy. Since the basal to middle lateral wall of left ventricle was usually involved in ALVC, we need to distinguish it from ischemic heart disease. When differentiating ALVC from myocardial infarction, the most important issue from our study is that ALVC has predominant subepicardial to endomyocardial involvement whereas myocardial infarction is just opposite (from endocardium to epicardium). Furthermore, the patients with ALVC also have the following additional characteristics: nearly all of the left ventricular inferolateral wall has fatty/fibro-fatty replacement and impaired systolic function, fatty/fibro-fatty replacement may also exist in other portions of the LV (basal ventricular septal intramural replacement), Holter/ECG suggest frequent ventricular arrhythmias origin from the LV, there is usually no angina or coronary artery disease, and finally, the most frequent presenting symptoms are usually palpitations and/or syncope.

### Limitations

Myocardial biopsy is considered the gold standard for the diagnosis of ARVC. However, due to the limitation of sample location and number, false negatives are a common problem^37,38^. In this cohort, the majority of the patients have disease within the LV rather than the RV, thus a RV biopsy was not an adequate means for evaluation. As a result, in this study we relied more on clinical/imaging criteria and heart transplantation to make the diagnosis of ALVC. Although MRI is crucial in the diagnosis of this disease, it still has intrinsic limitations in this study^39–41^. Finally, Genetic testing yields a pathogenic mutation in only 50% of patients. Classical genotype-phenotype correlation does exist, allowing early identification of the disease. However, there are still many common genetic mutations among various cardiomyopathy. There are still great uncertainties in using genotypes to determine phenotypes.

## Conclusions

In conclusion, ALVC is a rare disease with fibro-fatty replacement predominantly in the left ventricle, impaired left ventricular systolic function, and ventricular arrhythmias originating from the left ventricle. In comparison to ALVC patients without RV involvement, ALVC with RV involvement may have a worse prognosis, but this still needs to be further investigated.
